# Olanzapine as Antiemetic Prophylaxis in Moderately Emetogenic Chemotherapy: A Phase 3 Randomized Clinical Trial

**DOI:** 10.1001/jamanetworkopen.2024.26076

**Published:** 2024-08-06

**Authors:** Vikas Ostwal, Anant Ramaswamy, Sarika Mandavkar, Prabhat Bhargava, Deepali Naughane, Sharon Flavia Sunn, Sujay Srinivas, Akhil Kapoor, Bal Krishna Mishra, Anuj Gupta, Bipinesh Sansar, Vikash Pal, Aparajita Pandey, Avinash Bonda, Indraja Siripurapu, Vamshi Krishna Muddu, Sadhana Kannan, Deepali Chaugule, Rajshree Patil, Manali Parulekar, Aditya Dhanawat, Mehek Trikha, Jaya Ghosh, Vanita Noronha, Nandini Menon, Vijay Patil, Kumar Prabhash, Ian Olver

**Affiliations:** 1 Department of Medical Oncology, Tata Memorial Centre, Homi Bhabha National Institute, Mumbai, India; 2 Department of Medical Oncology, Homi Bhabha Cancer Hospital, Varanasi, Varanasi, India; 3 Medical Oncology, AIG Hospitals, Gachibowli, Hyderabad, India; 4 Department of Statistics, Advanced Centre for Treatment, Research and Education in Cancer, Homi Bhabha National Institute, Mumbai, India; 5 Department of Medical Oncology, PD Hinduja Hospital and Research Centre, Mumbai, India; 6 School of Psychology I Faculty of Health and Medical Sciences, University of Adelaide, Adelaide, Australia

## Abstract

**Importance:**

The role of olanzapine has not been adequately evaluated in moderately emetogenic chemotherapy (MEC) regimens with or without neurokinin-1 receptor antagonists.

**Objective:**

To evaluate whether addition of olanzapine to an MEC regimen reduces nausea, vomiting, and use of nausea rescue medications among patients with solid malignant tumors.

**Design, Setting, and Participants:**

This multicenter, open-label phase 3 randomized clinical trial included patients aged 18 years or older with solid malignant tumors who were receiving oxaliplatin-, carboplatin-, or irinotecan-based chemotherapy. The trial was conducted at 3 institutes in India from March 26, 2019, to August 26, 2023; the final cutoff date for analysis was September 10, 2023.

**Exposure:**

Patients were randomized 1:1 to dexamethasone, aprepitant, and palonosetron with olanzapine (experimental group) or without olanzapine (observation group). The experimental group received 10 mg of olanzapine orally once at night on days 1 through 3 of the chemotherapy regimen.

**Main Outcomes and Measures:**

The primary end point was complete response (CR), defined as the proportion of patients with no vomiting, no significant nausea (scored as <5 on a visual analog scale of 1 to 100), and no use of rescue medications for nausea. Secondary end points included the proportion of patients experiencing nausea and chemotherapy-induced nausea and vomiting (CINV), receiving rescue medications, and experiencing adverse events.

**Results:**

A total of 560 patients (259 [64%] male; median age, 51 years [range, 19-80 years]) were randomized. The analysis included 544 patients with evaluable data (274 assigned to olanzapine and 270 to observation). Baseline characteristics were evenly matched between the 2 groups. The proportion of patients with CR was significantly greater in the group with (248 [91%]) than without (222 [82%]) olanzapine in the overall 120-hour treatment period (*P* = .005). Likewise, there were significant differences between the olanzapine and observation groups for nausea control (264 [96%] vs 234 [87%]; *P* < .001) and CINV (262 [96%] vs 245 [91%]; *P* = .02) during the overall assessment period, and the proportion of patients receiving rescue medications significantly increased in the observation group (30 [11%]) compared with the olanzapine group (11 [4%]) (*P* = .001). Grade 1 somnolence was reported by 27 patients (10%) following administration of chemotherapy and olanzapine and by no patients in the observation group.

**Conclusions and Relevance:**

In this randomized clinical trial, the addition of olanzapine significantly improved CR rates as well as nausea and vomiting prevention rates in chemotherapy-naive patients who were receiving MEC regimens containing oxaliplatin, carboplatin, or irinotecan. These findings suggest that use of olanzapine should be considered as one of the standards of care in these chemotherapy regimens.

**Trial Registration:**

Clinical Trials Registry–India (CTRI) Identifier: CTRI/2018/12/016643

## Key Points

**Question:**

Does the addition of olanzapine improve rates of complete response (no vomiting, significant nausea, or use of rescue medications for nausea) among patients receiving moderately emetogenic chemotherapy (MEC)?

**Findings:**

In this phase 3, open-label, randomized clinical trial including 560 patients, the addition of olanzapine to dexamethasone, palonosetron, and aprepitant MEC regimens improved complete response rates from 82% to 91%.

**Meaning:**

The findings suggest that olanzapine should be considered as one of the standards of care to reduce chemotherapy-induced nausea and vomiting among patients receiving MEC.

## Teaser

This randomized clinical trial assesses whether addition of olanzapine reduces nausea, vomiting, and use of nausea rescue medications among patients with solid malignant tumors receiving moderately emetogenic chemotherapy.

## Introduction

Chemotherapy-induced nausea and vomiting (CINV) are troublesome adverse effects of chemotherapy that significantly impact the quality of life of patients undergoing cancer-directed therapy. Guideline-based, streamlined use of antiemetics has significantly improved CINV, but complete alleviation should remain the predominant goal.^1,2^

The traditional classification of potentially emetogenic antineoplastic agents recognizes oxaliplatin-, irinotecan-, and carboplatin-containing regimens as moderately emetogenic chemotherapy (MEC), with a 30% to 90% risk of emesis.^3^ The commonly used antiemetic guidelines have some differences with respect to the antiemetic prophylaxis (AEP) agent to be considered for oxaliplatin- and carboplatin-containing regimens.^2,4,5^ The European Society for Medical Oncology (ESMO) antiemetic guidelines suggest adding a neurokinin-1 receptor antagonist (NK-1RA) for women younger than 50 years receiving oxaliplatin-containing regimens and also set a cutoff of carboplatin (area under the receiver operating characteristic curve [AUC]≥5) for using a similar 3-drug AEP. In contrast, the American Society of Clinical Oncology guidelines set a cutoff of carboplatin (AUC≥4) for use of 3-drug AEP besides having no additional recommendations for use of oxaliplatin. The National Comprehensive Cancer Network guidelines also allow use of a 5-hydroxytryptamine type 3 (5-HT3) antagonist, dexamethasone, and olanzapine as AEP in MEC regimens. Patients receiving other MEC regimens need not receive an NK-1RA unless there are additional risk factors or AEP failure.^6,7^ However, clinical studies have shown significant breakthrough CINV rates with the use of these prophylactic regimens despite guideline-mandated use.^8^ This has led to trials evaluating the role of adding a prophylactic NK-1RA to all moderately emetogenic regimens.^9^

Olanzapine is an atypical antipsychotic agent that blocks multiple neurotransmitters, including dopamine, serotonin, catecholamines, histamine, and acetylcholine, in the central nervous system.^10^ It is approved as part of AEP for highly emetogenic chemotherapy based on phase 3 clinical trials.^11,12^ A small Korean study that added olanzapine to dexamethasone and palonosetron as AEP for MEC regimens also showed a decrease in nausea and use of rescue medications for nausea.^13^

Based on the philosophy of maximally reducing rates of CINV and the efficacy of olanzapine as AEP in highly emetogenic chemotherapy, the current trial evaluated whether olanzapine would improve complete response (CR) rates in MEC regimens comprising oxaliplatin, carboplatin (AUC≥5), or irinotecan.^11^ The study also assessed patient-reported outcomes using the Functional Living Index–Emesis (FLIE) questionnaire as a measure of efficacy of AEP.^14^

## Methods

### Study Design

This open-label, multicenter phase 3 randomized clinical trial was conducted from March 26, 2019, to August 26, 2023, at 3 institutes in India and involved patients receiving an MEC regimen. The institutional ethics committee at Tata Memorial Hospital approved the trial, and the other collaborating centers (Asian Institute of Gastroenterology, Hyderabad; Homi Bhabha Cancer Hospital and Mahamana Pandit Madan Mohan Malaviya Cancer Centre) obtained approval from their respective institutional ethics committees. The trial was registered prospectively with Clinical Trials Registry–India (CTRI/2018/12/016643). All patients gave written informed consent. The study was independently monitored by the data safety monitoring board of the Tata Memorial Hospital institutional ethics committee. This study followed the Consolidated Standards of Reporting Trials (CONSORT) reporting guideline.

### Eligibility Criteria

Patients aged 18 years or older who had solid malignant tumors and were chemotherapy naive were eligible for enrollment in the study if they were scheduled to receive MEC with capecitabine and oxaliplatin; leucovorin calcium, fluorouracil, and oxaliplatin 7; pemetrexed plus carboplatin (AUC≥5); paclitaxel plus carboplatin (AUC≥5); folinic acid, fluorouracil, and irinotecan hydrochloride; capecitabine and irinotecan hydrochloride; or gemcitabine and oxaliplatin and had an Eastern Cooperative Oncology Group Performance Status Scale score of 0 or 1 (on a 6-point scale). Major exclusion criteria were emesis or clinically significant nausea (defined as nausea graded as moderate or severe) in the 24 hours preceding the first dose of study medication, uncontrolled comorbidities, or known hypersensitivity or contraindication to the drugs used as AEP or receipt of treatment that may have influenced medications used in the study. The complete trial inclusion and exclusion criteria are provided in the trial protocol in Supplement 1.

### Study Design and Oversight

Patients underwent simple permuted block randomization in a 1:1 ratio with a block size of 2 or 4 to the observation group or the olanzapine group using a computer-generated list. There were no stratification factors used between the study groups. The study was blinded at 2 levels. The medical professionals involved in the treatment of patients and the trial coordinator (S.M.) assessing the degree of nausea, vomiting, and use of rescue medications were unaware of the assigned treatment arm. The statistician was also blinded to the assigned arms of the study while analyzing the results. Patients were aware of whether they were receiving olanzapine or not and were not blinded. The primary end point was assessment of CR rates in the first cycle of chemotherapy (0-120 hours). Receipt of rescue therapy for nausea or vomiting was allowed as per the treating physician’s choice.

### Treatment Regimen

All participants received palonosetron (0.25 mg intravenously on day 1 of chemotherapy), dexamethasone (12 mg intravenously on day 1), and aprepitant (125 mg orally on day 1 and 80 mg orally on days 2 and 3). In the experimental arm, patients additionally received olanzapine (10 mg per day orally once at night) on days 1 through 3.

### Study Visits and Assessment Procedures

Demographic characteristics and baseline clinical data for all patients were collected prior to enrollment in the study. A diary was provided to patients to record their intake of medications related to antiemetic prophylaxis, episodes of vomiting or nausea, and whether medications were used for rescue. Patients were also asked to record daily levels of nausea according to a visual analog scale ranging from 0 (no nausea at all) to 100 (nausea as bad as it can be). The diary included reminders with regard to the scheduling of antiemetic drugs. A dedicated study nurse (M.P.) contacted each patient twice daily on days 1 through 5 to inquire about and document toxic effects. Patients were questioned on whether they experienced increased somnolence during days 2 to 7 by the study nurse, and this was classified based on the Common Terminology Criteria for Adverse Events, version 5.0 for somnolence. Quality of life (QOL) assessments were conducted using the Functional Living Index–Emesis (FLIE) questionnaire at baseline and 7 to 10 days after administration of the first cycle of chemotherapy.

### Outcomes

The primary end point of the study was the CR rate, defined as a response of less than 5 on the visual analog scale for nausea and no vomiting or use of rescue medications during the overall assessment period of 0 to 120 hours in the first cycle of chemotherapy. Secondary end points included assessments of CR during the early (0-24 hours) and late (25-120 hours) assessment periods; nausea, vomiting, and CINV individually during the overall assessment period of 0 to 120 hours and during the early and late assessment periods; efficacy of the carboplatin- and oxaliplatin-containing regimens in both arms; and CR rates, nausea control, vomiting control, and CINV control rates cumulatively during the second and third cycles of chemotherapy. Nausea control was defined as a score less than 5 on the visual analog scale for nausea, vomiting control was defined as absence of vomiting in the prespecified period, and CINV control was defined as absence of nausea (as previously defined) or vomiting during the prespecified period. Adverse events of all grades were noted in both arms of the study. After the accrual of the first 45 patients in the study, it was decided to additionally evaluate the CINV risk assessment model^15^ to identify whether the emetic risk in patients during the first cycle of chemotherapy could be quantified using patient-related factors beyond the traditional chemotherapy-based risk stratification.^16,17^ Patients were classified as having low or high CINV risk based on the results of the risk assessment tool, although no changes were made to treatment based on the risk assessment. The CR and CINV rates were calculated individually in these 2 groups and were stratified for the olanzapine and observation groups.

### Statistical Analysis

The primary end point of CR rate was compared between the treatment groups using χ^2^ tests initially for the overall period and then for the early and later periods separately. It was assumed that the observation arm in the study would have a CR rate of 75% based on extrapolation from available data, as CR rates with NK-1RA–based combinations have ranged between 69% and 85%.^9,13,18^ Based on this assumption, 560 patients (280 per arm) were required to show an improvement in CR rates of 10% (ie, CR rate of 85%) in the arm with the olanzapine-containing regimen. This was based on a 2-sided α of 0.05 and power of 80%, assuming 10% attrition rates. The sample size calculation was conducted with the use of an online sample size calculator.^19^

Secondary end points were compared using χ^2^ tests, and reported *P* values for these analyses were not adjusted for multiple comparisons. The QOL assessment using FLIE was conducted using 2 methods. The first method was based on inputs from the study statistician and performed by dividing the patient groups based on score deterioration by more than 5 from the baseline score for nausea or vomiting and more than 10 from the baseline score for combined CINV. The 2 groups were compared by the χ^2^ method for the significance of differences. The second method was based on interpretation of the FLIE scores as used in a randomized clinical trial evaluating antiemetic therapy for patients with breast cancer receiving chemotherapy.^20^ This method involved assigning the FLIE scores to a visual analog scale from 0 to 100 and dividing them as follows: 0 to 5 (no nausea), 6 to 25 (no significant nausea), and more than 25 (significant nausea).^20^ Similar cutoffs were used for the measurement of vomiting and of nausea and vomiting combined. We compared the proportions of patients in the observation and olanzapine groups scoring 0 to 5 for nausea, vomiting, and both nausea and vomiting by the χ^2^ method. The final cutoff date for analysis was September 10, 2023. Analyses were conducted with SPSS, version 25 (SPSS Institute Inc).

## Results

### Study Patients

**Figure 1.**
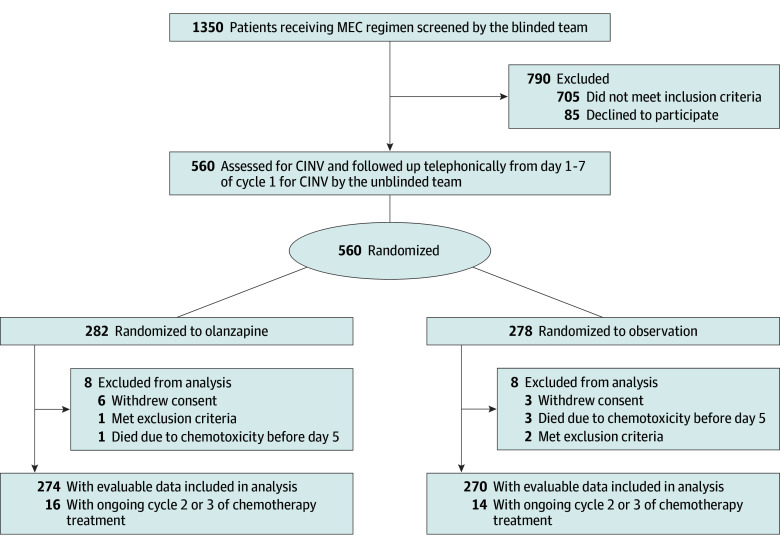
CONSORT Diagram CINV indicates chemotherapy-induced nausea and vomiting; MEC, moderately emetogenic chemotherapy.

Figure 1 shows the distribution and randomization of patients in the study. A total of 560 patients (201 [36%] female and 259 [64%] male; median age, 51 years [range, 19-80 years]) were randomly assigned to a study group (282 to olanzapine and 278 to observation). All 560 patients began the study, and 544 (274 [97%] assigned to olanzapine and 270 [97%] to observation) were analyzed for end points in the study. Baseline characteristics in the 2 groups were well balanced and are presented in Table 1.

**Table 1.** Baseline Demographic and Clinical Characteristics of the Study Patients

Characteristic	Patients, No. (%)		
	Olanzapine (n = 282)	Observation (n = 278)	Total (N = 560)
Age, median (range), y	51 (20-80)	50 (19-78)	51 (19-80)
Sex			
Female	102 (36)	99 (36)	201 (36)
Male	180 (64)	179 (64)	359 (64)
Chemotherapy regimen			
Oxaliplatin-containing regimen	172 (61)	169 (61)	341 (61)
Irinotecan-containing regimen	31 (11)	23 (8)	54 (10)
Carboplatin-containing regimen	79 (28)	86 (31)	165 (29)
Primary site of disease			
Colorectal	165 (59)	159 (57)	324 (58)
Gastric or gastroesophageal	22 (8)	22 (8)	44 (8)
Non–small cell lung carcinoma	26 (9)	29 (10)	55 (10)
Biliary tract carcinoma	31 (11)	30 (11)	61 (11)
Urinary bladder cancer	24 (9)	24 (9)	48 (9)
Others	12 (4)	14 (5)	26 (5)
Cancer stage			
II	13 (5)	14 (5)	27 (5)
III	124 (44)	126 (45)	250 (45)
IV	145 (51)	138 (50)	283 (51)
CINV risk score			
Low	202 (72)	180 (65)	382 (68)
High	60 (21)	73 (26)	133 (24)
Not available	20 (7)	25 (9)	45 (8)

Abbreviation: CINV, chemotherapy-induced nausea and vomiting.

### Efficacy

**Figure 2.**
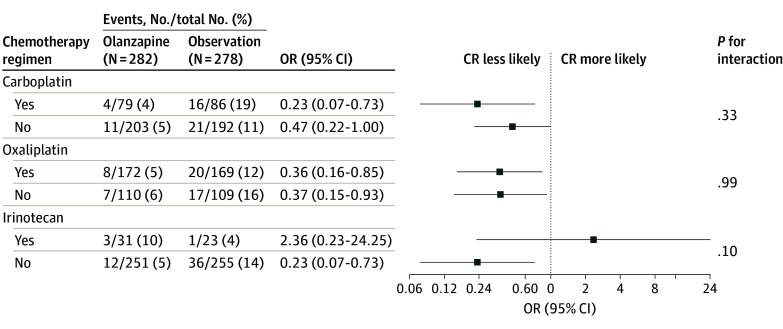
Test of Interactions on Complete Response (CR) Rates and Type of Chemotherapy Regimen OR indicates odds ratio.

At the final cutoff date for analysis, the proportion of patients who had experienced CR (the primary end point) was significantly greater in the olanzapine group than in the observation group during the overall assessment period (0-120 hours) (248 [91%] vs 222 [82%]; *P* = .005) and the later period (25-120 hours) (253 [92%] vs 233 [83%]; *P* = .001). The differences in CR during the early period (0-24 hours) were not significant (olanzapine, 262 [96%]; observation, 255 [94%]; *P* = .53) (Table 2). On subgroup analysis based on the type of chemotherapy, CR in the olanzapine arm was noted in a greater proportion of patients receiving oxaliplatin-based chemotherapy (odds ratio [OR], 0.36 (95% CI, 0.16-0.85) and carboplatin-based chemotherapy (OR, 0.23; 95% CI, 0.07-0.73) but not irinotecan-based therapy (OR, 2.36; 95% CI, 0.23-24.25) (Figure 2).

**Table 2.** Primary End Point According to Study Group

Variable	Patients, No. (%)			*P* value^a^
	Olanzapine (n = 274)	Observation (n = 270)	Total (N = 544)	
**0-120 h After chemotherapy**				
Complete response	248 (91)	222 (82)	470 (86)	.005
Absence of complete response	26 (9)	48 (18)	54 (14)	
**0-24 h After chemotherapy**				
Complete response	262 (96)	255 (94)	517 (95)	.53
Absence of complete response	12 (4)	15 (6)	27 (5)	
**25-120 h After chemotherapy**				
Complete response	253 (92)	233 (83)	486 (89)	.001
Absence of complete response	21 (8)	37 (17)	58 (10)	

^a^ *P* values were obtained by χ^2^ test.

Differences between the olanzapine and observation groups with respect to important secondary end points as well as during the second and third cycles of chemotherapy are shown in Table 3 and eTables 1 and 2 in Supplement 2, respectively. There were significant differences between the olanzapine and observation groups for nausea control (264 [96%] vs 234 [87%]; *P* < .001) and CINV control (262 [96%] vs 245 [91%]; *P* = .02) during the overall assessment period. The proportion of patients receiving rescue medications was significantly increased in the observation group (30 [11%]) compared with the olanzapine group (11 [4%]) (*P* = .001).

**Table 3.** Detailed Analysis of Secondary End Points According to Study Group

Variable	Patients, No. (%)		*P* value^a^
	Olanzapine group (n = 274)	Observation group (n = 270)	
**Overall**			
Nausea control	264 (96)	234 (87)	<.001
Vomiting control	261 (95)	255 (94)	.67
CINV control	262 (96)	245 (91)	.02
**Early**			
Nausea control	267 (97)	257 (95)	.16
Vomiting control	267 (97)	266 (99)	.37
CINV control	262 (96)	255 (94)	.53
**Delayed**			
Nausea control	265 (97)	239 (89)	<.001
Vomiting control	265 (97)	257 (95)	.37
CINV control	261 (95)	233 (86)	<.001

Abbreviation: CINV, chemotherapy-induced nausea and vomiting.

^a^ *P* values were obtained by the χ^2^ test.

### Exploratory Analysis of End Points According to CINV Risk Score

Risk stratification according to the CINV risk score was feasible for 515 patients (92%). In the overall population, there were no significant differences between the low-risk and high-risk groups in terms of CR rates (336 [88%] vs 113 [85%]; *P* = .17) and CINV control rates (344 [90%] vs 117 [88%]; *P* = .51).

Patients in the olanzapine group classified as low risk had significantly increased CR rates compared with patients in the observation group classified as low risk (184 [91%] vs 151 [84%]; *P* = .03), while there were no differences between the olanzapine and observation groups in patients classified as high risk (53 [88%] vs 60 [82%]; *P* = .32). Similarly, patients in the olanzapine group classified as low risk had significantly increased CINV control rates compared with patients in the observation group classified as low risk (190 [94%] vs 152 [84%]; *P* = .002), while there were no differences in the olanzapine and observation groups in patients classified as high risk (55 [92%] vs 62 [85%]; *P* = .24) (eTable 3 in Supplement 2).

### Adverse Events and Tolerance

Grade 1 somnolence was reported by 27 patients (10%) following administration of chemotherapy and olanzapine and by no patients in the observation arm. There were no other adverse events that were deemed related to olanzapine in the study.

### QOL

Reduced QOL, as measured by a FLIE scale score reduction of more than 10 points from baseline, occurred in 31 patients (12%) in the observation arm compared with 17 (6%) in the olanzapine arm (*P* = .03) when they were assessed for CINV, and it was reduced by more than 5 points in 22 patients (8%) in the observation arm compared with 9 (3%) in the olanzapine arm when they were assessed for vomiting (*P* = .01). There was no significant difference in deterioration in nausea-related QOL (33 patients [12%] in the observation arm vs 26 [9%] in the olanzapine arm; *P* = .30). Using the second method of QOL assessment by FLIE based on classifying patients with scores of 0 to 5 as having no nausea, vomiting, or nausea and vomiting, there were no differences between the observation and olanzapine arms in terms of nausea control (240 [89%] vs 248 [91%]; *P* = .53) and vomiting control (251 [93%] vs 266 [97%]; *P* = .07) individually. However, significantly greater control in terms of FLIE scores was noted in the olanzapine arm when nausea and vomiting were considered together (258 [94%] vs 238 [88%]; *P* = .02)

## Discussion

This phase 3 randomized clinical trial showed that adding olanzapine to a combination of aprepitant, palonosetron, and dexamethasone improved CR rates, nausea control, and control of CINV in chemotherapy-naive patients receiving MEC regimens comprising oxaliplatin, carboplatin, or irinotecan. The addition of NK-1RAs to MEC regimens is controversial, barring the notable exception of carboplatin (AUC≥4 or ≥5), based on a phase 3 trial evaluating rolapitant^6^ and a post hoc analysis of a phase 3 trial evaluating aprepitant in the control of hypersensitivity reactions with paclitaxel.^7^ The phase 3 SENRI trial showed a similar benefit with oxaliplatin-containing regimens,^18^ although a later meta-analysis questioned the benefits of adding NK-1RAs to MEC regimens as a whole.^9^ A deeper evaluation of those trials showed that CR was not achieved in 15% to 20% of patients receiving an NK-1RA and 25% to 35% of those not receiving an NK-1RA. Considering that CINV is a major problem with no positive inferences or benefits for patients, allowing such high CINV rates should not be acceptable. Additionally, if there are efficacious, safe, and cost-effective medications (such as olanzapine) to ensure high CR rates, these medications should be considered for increased patient benefit and evaluated in trials. Additionally, the Korean South West Oncology Group study provided early evidence to explore the use of olanzapine in MEC regimens.^13^ That study, however, was underpowered (N = 56) and did not use NK-1RAs as AEP, thereby suggesting a need for conduct of a study using a 4-drug AEP in MEC regimens.

The current study achieved higher than expected CR rates in both arms, with the addition of olanzapine improving CR rates by 9%. The effect of olanzapine appeared to predominantly occur in the delayed phase of CINV prevention, as evinced by the improvement across end points (CR, CINV, and nausea) in the delayed phase.^12^ A key parameter that is commonly used by major guidelines as an assessment in improving AEP is whether an intervention improves an emesis-related end point by 10% or more.^1^ While the improvement in CR rates with the addition of olanzapine in this study fell marginally short of this conventional arbitrary margin, the improvement across end points, lesser use of rescue medications, negligible adverse effect profile, and improvement in QOL as measured by the FLIE scores support the addition of olanzapine as AEP in patients receiving oxaliplatin, carboplatin, or irinotecan.

For the first time to our knowledge, the CINV risk score was prospectively evaluated in this study, and the results are hypothesis generating. First, most patients in the study were classified as low risk as opposed to high risk for CINV, and this could be one of the reasons for the higher than expected overall CR rates in the study. Second, the additive effect of olanzapine in improving CR rates and reducing CINV rates was greater among patients at low risk than among those at high risk. Reasons for this variance could be that the score recognizes the standard antiemetic regimen for non–carboplatin-containing MEC as a combination of a 5-HT3 antagonist and dexamethasone and calculates the baseline CINV risk based on the efficacy of this combination, whereas the current study used an additional NK-1RA as a baseline comparator.

The improvements seen in CR rates with the addition of olanzapine were also reflected by QOL assessments using the FLIE questionnaire. The statistically significant deterioration in QOL in the observation arm compared with the olanzapine arm when assessed for CINV added to the benefits achieved in our trial.

### Limitations

One of the major limitations of our study is that it did not use a placebo in the control arm. Other limitations included the use of a single-dose regimen for olanzapine (10 mg) and no assessment of the efficacy of a 5-mg dose regimen. We did not report other effects seen with olanzapine, such as increase in appetite or constipation, although grade 3 or 4 events with respect to these effects were not noted. There was also a predominance of patients with gastrointestinal cancers receiving oxaliplatin-containing regimens as opposed to other primary cancers and regimens.

## Conclusions

In this randomized clinical trial, olanzapine, 10 mg, combined with aprepitant, palonosetron, and dexamethasone, improved CR rates compared with no olanzapine. These findings suggest that this regimen could be considered as one of the standards of antiemetic therapy in patients receiving oxaliplatin-, irinotecan-, or carboplatin-based chemotherapy. The use of the CINV risk score should be explored further when making treatment decisions for using AEP in these MEC regimens.

## Notes

Supplement 1.
Trial Protocol

Supplement 2. **eTable 1.** End Points by Frequencies and Proportions According to the Study Group in the Second Cycle of Chemotherapy **eTable 2.** End Points by Frequency and Proportions According to the Study Group in the Third Cycle of Chemotherapy **eTable 3.** End Points According to Study Group and CINV Risk Score

Supplement 3.
Data Sharing Statement

## References

[1] Roila F, Molassiotis A, Herrstedt J, ; participants of the MASCC/ESMO Consensus Conference Copenhagen 2015. 2016 MASCC and ESMO guideline update for the prevention of chemotherapy- and radiotherapy-induced nausea and vomiting and of nausea and vomiting in advanced cancer patients. Ann Oncol. 2016;27(suppl 5):v119-v133. doi:10.1093/annonc/mdw270 27664248

[2] Hesketh PJ, Kris MG, Basch E, . Antiemetics: American Society of Clinical Oncology Clinical Practice Guideline Update. J Clin Oncol. 2017;35(28):3240-3261. doi:10.1200/JCO.2017.74.4789 28759346

[3] Hesketh PJ, Kris MG, Grunberg SM, . Proposal for classifying the acute emetogenicity of cancer chemotherapy. J Clin Oncol. 1997;15(1):103-109. doi:10.1200/JCO.1997.15.1.103 8996130

[4] Herrstedt J, Clark-Snow R, Ruhlmann CH, ; participants of the MASCC/ESMO Consensus Conference 2022. 2023 MASCC and ESMO guideline update for the prevention of chemotherapy- and radiotherapy-induced nausea and vomiting. ESMO Open. 2024;9(2):102195. doi:10.1016/j.esmoop.2023.102195 38458657 PMC10937211

[5] Kennedy SKF, Goodall S, Lee SF, et al. 2020 ASCO, 2023 NCCN, 2023 MASCC/ESMO, and 2019 CCO: a comparison of antiemetic guidelines for the treatment of chemotherapy-induced nausea and vomiting in cancer patients. *Support Care Cancer*. 2024;32(5):280. doi:10.1007/s00520-024-08462-x 38594320

[6] Hesketh PJ, Schnadig ID, Schwartzberg LS, . Efficacy of the neurokinin-1 receptor antagonist rolapitant in preventing nausea and vomiting in patients receiving carboplatin-based chemotherapy. Cancer. 2016;122(15):2418-2425. doi:10.1002/cncr.30054 27176138 PMC5084806

[7] Yahata H, Kobayashi H, Sonoda K, . Efficacy of aprepitant for the prevention of chemotherapy-induced nausea and vomiting with a moderately emetogenic chemotherapy regimen: a multicenter, placebo-controlled, double-blind, randomized study in patients with gynecologic cancer receiving paclitaxel and carboplatin. Int J Clin Oncol. 2016;21(3):491-497. doi:10.1007/s10147-015-0928-y 26662632

[8] D’Souza A, Pawar D, Ramaswamy A, . Chemotherapy-induced nausea and vomiting (CINV) with GI cancer chemotherapy: do we need CINV risk score over and above antiemetic guidelines in prescribing antiemetic regime? South Asian J Cancer. 2020;9(4):240-244. doi:10.1055/s-0041-1726136 34131576 PMC8197652

[9] Jordan K, Blättermann L, Hinke A, Müller-Tidow C, Jahn F. Is the addition of a neurokinin-1 receptor antagonist beneficial in moderately emetogenic chemotherapy? A systematic review and meta-analysis. Support Care Cancer. 2018;26(1):21-32. doi:10.1007/s00520-017-3857-7 28861627

[10] Navari RM. Olanzapine for the prevention and treatment of chronic nausea and chemotherapy-induced nausea and vomiting. Eur J Pharmacol. 2014;722:180-186. doi:10.1016/j.ejphar.2013.08.048 24157985

[11] Navari RM, Qin R, Ruddy KJ, . Olanzapine for the prevention of chemotherapy-induced nausea and vomiting. N Engl J Med. 2016;375(2):134-142. doi:10.1056/NEJMoa1515725 27410922 PMC5344450

[12] Hashimoto H, Abe M, Tokuyama O, . Olanzapine 5 mg plus standard antiemetic therapy for the prevention of chemotherapy-induced nausea and vomiting (J-FORCE): a multicentre, randomised, double-blind, placebo-controlled, phase 3 trial. Lancet Oncol. 2020;21(2):242-249. doi:10.1016/S1470-2045(19)30678-3 31838011

[13] Jeon SY, Han HS, Bae WK, . A randomized, double-blind, placebo-controlled study of the safety and efficacy of olanzapine for the prevention of chemotherapy-induced nausea and vomiting in patients receiving moderately emetogenic chemotherapy: results of the Korean South West Oncology Group (KSWOG) study. Cancer Res Treat. 2019;51(1):90-97. doi:10.4143/crt.2017.577 29510613 PMC6333980

[14] Martin AR, Carides AD, Pearson JD, . Functional relevance of antiemetic control. Experience using the FLIE questionnaire in a randomised study of the NK-1 antagonist aprepitant. Eur J Cancer. 2003;39(10):1395-1401. doi:10.1016/S0959-8049(03)00299-5 12826042

[15] CINV risk assessment. Accessed September 16, 2023. https://www.riskcinv.org/#/

[16] Molassiotis A, Stamataki Z, Kontopantelis E. Development and preliminary validation of a risk prediction model for chemotherapy-related nausea and vomiting. Support Care Cancer. 2013;21(10):2759-2767. doi:10.1007/s00520-013-1843-2 23715816

[17] Dranitsaris G, Molassiotis A, Clemons M, . The development of a prediction tool to identify cancer patients at high risk for chemotherapy-induced nausea and vomiting. Ann Oncol. 2017;28(6):1260-1267. doi:10.1093/annonc/mdx100 28398530 PMC5452068

[18] Nishimura J, Satoh T, Fukunaga M, ; Multi-center Clinical Study Group of Osaka, Colorectal Cancer Treatment Group (MCSGO). Combination antiemetic therapy with aprepitant/fosaprepitant in patients with colorectal cancer receiving oxaliplatin-based chemotherapy (SENRI trial): a multicentre, randomised, controlled phase 3 trial. Eur J Cancer. 2015;51(10):1274-1282. doi:10.1016/j.ejca.2015.03.024 25922233

[19] Clin Calc LLC. Sample size calculator. July 24, 2019. Accessed September 12, 2023. https://clincalc.com/stats/samplesize.aspx

[20] Clemons M, Dranitsaris G, Sienkiewicz M, . A randomized trial of individualized versus standard of care antiemetic therapy for breast cancer patients at high risk for chemotherapy-induced nausea and vomiting. Breast. 2020;54:278-285. doi:10.1016/j.breast.2020.11.002 33242754 PMC7695916

