# Supplementary material for: Olanzapine as Antiemetic Prophylaxis in Moderately Emetogenic Chemotherapy: A Phase 3 Randomized Clinical Trial
Source: JAMA Netw Open. 2024 Aug 6;7(8):e2426076. doi: 10.1001/jamanetworkopen.2024.26076 (PMC11304110; doi:10.1001/jamanetworkopen.2024.26076)
Supplement: Supplement 1. — Trial Protocol [file jamanetwopen-e2426076-s001.pdf]

Table of contents

Serial number	Components	Page number
1	Protocol title	
2	Study rationale	
3	Objectives and hypothesis	
	I.Primary	
	II.Secondary	
4	Study design	
	I.Study design	
	II.Treatment plan	
	III.Dosing schema	
5	Study medications	
	1. Olanzapine	
	I.Rationale for dosage of Olanzapine	
	II.Potential adverse reactions of Olanzapine	
	2. Aprepitant	
	I.Rationale for dosage of Aprepitant	

	II.Potential adverse reactions of Aprepitant	
	3. Dexamethasone	
	I.Rationale for dosage of Dexamethasone	
	II.Potential adverse reactions of Dexamethasone	
	4. Palonosetron	
	I.Rationale for dosage of Palonosetron	
	II.Potential adverse reactions of Palonosetron	
6	Randomization	
7	I. Preparation of medicines (antiemetics) for administration	
	• Aprepitant	
	• Dexamethasone	
	• Olanzapine	
	• Palonosetron	
	II. Handling and storage	
8	Documentation	

9	Assessment of compliance	
10	Study closure	
	I. Study closure	
	II. Withdrawal criteria	
	III. Source documents	
11	Study population	
	I.Screening visit, patient information, informed consent	
	II.Patient inclusion criteria	
	III.Patient exclusion criteria	
	IV.Screen and baseline failures	
11	Study assessments and procedures	
	I. Demographic and baseline assessments	
	II.Assessments in cycle 1	
	• Cycle 1 Day 1 visit	
	• Cycle 1 Day 2 - day 3 plan	
	• Cycle 1 mid-cycle evaluation (D8-D10)	

	● Cycle 1 End of cycle visit and assessments in cycles 2-3	
	III. Follow up visit and subject completion	
	IV. Antiemetic rescue medications	
	● Definition of rescue medications	
	● Permissible rescue medications	
	V. Antiemetic treatment following study treatment failure	
12	Safety	
13	Assessment of efficacy and tolerability	
	I. Efficacy measurements	
	● Patient diary	
	● Telephonic information	
	II. Nausea scales	
	III. Tolerability measurements	
	● Adverse events and serious adverse events	
	● Definition of AE	
	● Definition of SAE	

	● Definition of AR, SAR, SUSAR	
14	Disease related events and or disease related outcomes not qualifying as SAE/SAR	
15	Laboratory abnormalities and other abnormal assessments as AEs/ARs and SAEs/SARs	
16	Recording and reporting of AE, AR, SAE, SAR, and SUSAR	
17	Quality of life measurement	
	● Functional Living Index-Emesis (FLIE)	
18	Concomitant treatment	
	I.Chemotherapy	
	II.Prohibited medications	
19	Data analysis	
	I. Hypothesis	
	II. Study design and statistical considerations	
	III.Data analysis Considerations	
	IV.Treatment endpoints - primary and secondary	
20	Analytical plan	

	IAssessment windows	
	II.Efficacy analysis	
	III Tolerability analysis	
	IVStatistical deviations	
21	Conduct of study	
	I. Ethical considerations	
	II.Records maintenance	
	III.Data management	
	IV. Missing data	
22	Financing and assurances	
23	Publications	
24	References	
25	Appendices	

1. Protocol title

A randomized, open label, parallel-group, phase 3 study to investigate the efficacy and tolerability of palonosetron, dexamethasone, aprepitant plus Olanzapine versus palonosetron, dexamethasone and aprepitant alone in patients receiving high moderately emetogenic chemotherapeutic (MEC) regimens (OMEC study)

2. Study rationale

Chemotherapy induced nausea and vomiting (CINV) are unpleasant and worrisome side effects associated with the administration of chemotherapy. Improved control of emesis positively impacts quality of life in patients receiving chemotherapy and lack of adequate control also reflects adversely on quality of life (1). While current antiemetic regimens have markedly reduced the incidences of vomiting across chemotherapy regimens, control of nausea has not been as effective with lesser importance also having been given to this equally important and distressing side effect(2,3).

The term ‘Moderately emetogenic chemotherapy (MEC)’ is a mixed bag of regimens with varying incidences of CINV (30%-90%) with currently used antiemetic regimens. The currently approved antiemetic regimen for MEC is the combination of a 5 HT3 antagonist and dexamethasone, with the exception of regimens using carboplatin AUC >4, where the addition of a neurokinin (NK)1-receptor antagonist (RA) is recommended(4,5). Besides Carboplatin, other chemotherapeutic agents which may be considered to have high to moderate emetogenic potential include Irinotecan and Irinotecan based combination regimens(6).

The addition of NK1-RA antagonists has improved complete response rates in HEC and MEC regimens - however, 20% - 38% of patients still do not achieve complete response (CR)(7,8). Beyond not achieving CR, a majority of the seminal clinical trials have not concentrated on control of nausea. While acute, and to a certain extent delayed vomiting control rates have improved, control of nausea still remains low. The lack of control of nausea is one of the major reasons for a lag between control of vomiting and CR rates for nausea and vomiting.

Olanzapine is one of the recommended drugs for use in highly emetogenic chemotherapy (HEC) regimens (9,10). Known as an atypical antipsychotic agent of the thiobenzo-diazepine class, olanzapine was approved by the USA FDA (Food and Drug Administration) for the treatment of the manifestations of psychotic disorders in 1996. Olanzapine blocks multiple neurotransmitter receptors including dopaminergic D1, D2, D3,

D4 brain receptors, serotonergic 5-HT2a, 5-HT2c, 5-HT3, 5-HT6 receptors, catecholamine alpha1 adrenergic receptors, acetylcholine muscarinic receptors, and histamine H1 receptors. Moreover, olanzapine may reduce opioid requirements in cancer patients with uncontrolled pain, cognitive impairment, or anxiety. Due to the broad and potent inhibitory activity of olanzapine at multiple receptors involved in the nausea and vomiting pathways, this agent is an effective treatment as well as prophylaxis for CINV. It is also a significant inhibitory effect on nausea from available data. Besides its efficacy in CINV, it is also a low cost medication with a tolerable safety profile. Side effects may include mild short-term sedation, as well as weight gain and an

increased risk of diabetes mellitus with prolonged use (>6 months). The efficacy of Olanzapine has been shown in small Phase 2 and retrospective studies when used as an additional antiemetic in MEC(11–13). However, there is no phase 3 study examining the role of olanzapine in MEC.

The purpose of this study is therefore to investigate in a randomized manner, if the addition of Olanzapine to the combination of a 5-HT3-RA (palonosetron) plus a corticosteroid (dexamethasone) and NK_1-RA improves the antiemetic efficacy in patients receiving specific 'high' MEC regimens.

The assessment of quality of life in cancer patients as per patient reported outcomes of a treatment sometimes differ in comparison with the clinical objective outcome. Keeping this in mind, we will also investigate the patient reported outcomes with respect to nausea and vomiting. As part of this, the Functional Living-Index Emesis (FLIE) questionnaire will be used as part of the study.

3. Objectives and hypothesis

I. Primary

Objectives: The primary objective is to compare an antiemetic regimen consisting of aprepitant, palonosetron, dexamethasone and olanzapine (active arm) and a regimen consisting of aprepitant, palonosetron, and dexamethasone, (control arm) with respect to complete response (CR); the proportion of subjects with no vomiting, no significant nausea (scored as < 5 on a scale of 1-100) and no use of rescue medications during pre-specified high MEC protocols for 1 cycle of chemotherapy

Hypothesis: The addition of Olanzapine to palonosetron, dexamethasone and aprepitant combination will increase the CR rates [(the proportion of subjects with no vomiting, no significant nausea (scored as < 5 on a scale of 1-100) and no use of rescue medications] during high MEC regimens.

II. Secondary

Objectives

1. To compare the olanzapine containing regimen to the control arm with respect to no emesis rates (the proportion of subjects with no vomiting, and no use of rescue medications) during pre-specified high MEC protocols for 1 and 3 cycles of chemotherapy

2.to compare the olanzapine containing regimen to the control arm with respect to proportion of patients with no significant nausea (< 5 on a score of 1- 100) during pre-specified high MEC protocols for 1 to 3 cycles of chemotherapy

3. To compare the olanzapine containing regimen to the control arm with respect to CR rates during pre-specified high MEC protocols for the second and third cycles of chemotherapy
4. to compare quality of life using FLIE questionnaire
5. To compare tolerance and side effects with both regimens

4. STUDY DESIGN

I. Study design

This is a single institution, randomized, open label parallel-group study to investigate the efficacy and tolerability of palonosetron, dexamethasone and aprepitant with or without olanzapine in patients receiving 'high' MEC chemotherapy regimens (as specified below). Patients should not have received prior chemotherapy at any point of time previously (chemotherapy naive). The specific regimens received by patients and eligible for inclusion in study are -

1. Modified FOLFIRI (5 Fluorouracil + Leucovorin + Irinotecan) (biweekly)
   5 Fluorouracil 2400 mg/m2 intravenous infusion over 46-48 hours (diluted in 0.9% normal saline (NS)
   Irinotecan 180 mg/m2 intravenous infusion over 90 minutes (diluted in 0.9% normal Saline or D5W)
   Leucovorin 400 mg intravenous over 2 hours (prior to 5 Fluorouracil infusion)
2. CAPIRI (Capecitabine + Irinotecan) (every 3 weeks)
   Tablet Capecitabine 1700 mg/m2/day in two divided doses per oral for 14 days, then 7 Days off
   Irinotecan 200 mg/m2 intravenous infusion over 90 minutes (diluted in 0.9% normal Saline or D5W)
3. CAPOX (Capecitabine + Oxaliplatin) (every 3 weeks)
   Tablet Capecitabine 2000 mg/m2/day in two divided doses per oral for 14 days, then 7 days off
   Oxaliplatin 130 mg/m2 intravenous infusion over 120 - 360 minutes (diluted in D5W)
4. Modified FOLFOX (5 Fluorouracil + Leucovorin + Oxaliplatin) (biweekly)
   5 Fluorouracil 2400 mg/m2 intravenous infusion over 46-48 hours (diluted in 0.9% normal saline (NS)
   Oxaliplatin 85 mg/m2 intravenous infusion over 120 - 360 minutes (diluted in D5W)
   Leucovorin 400 mg intravenous over 2 hours (prior to 5 Fluorouracil infusion)
5. Pemetrexed plus Carboplatin (every 3 weeks)

Pemetrexed 500 mg/m2 intravenous over 10 mins (diluted in 0.9% normal saline)
Carboplatin (AUC - 5 or AUC - 6) intravenous over 30 minutes

6. Paclitaxel plus Carboplatin (every 3 weeks)
   Paclitaxel 175 - 225 mg/m2 intravenous over 3 hours (diluted in 0.9% normal saline)
   Carboplatin (AUC - 5 or AUC - 6) intravenous over 30 minutes
7. GEMOX (Gemcitabine + Oxaliplatin) (every 2 weeks)
   Gemcitabine - 1000mg/m2 intravenous over 30 minutes (diluted in 0.9% normal saline)
   Oxaliplatin 85 mg/m2 intravenous infusion over 120 - 360 minutes (diluted in D5W)

A total of 560 eligible patients will be randomized into 1 of 2 treatment arms and receive either a four drug antiemetic regimen consisting of palonosetron, dexamethasone, aprepitant and olanzapine or a three drug antiemetic regimen consisting of palonosetron, dexamethasone and aprepitant. This will yield approximately 560 evaluable study subjects.

Eligible patients will be block randomized to the two treatment arms. Subjects will be assigned to one of 2 treatment regimens according to an allocation schedule of random numbers.

The study will start after IEC/IRB approval. Based on the number of patients seen in Gastrointestinal Medical Oncology, Thoracic Medical Oncology, Head and Neck Medical Oncology and Urological Medical Oncology units, we expect to complete accrual of planned number of patients in approximately 36 months. A follow up period of 6 months will be required for completion of study post accrual.

The study will be conducted in accordance with this protocol and applicable regulatory requirements.

II. TREATMENT PLAN

The time and sequence of drug administration in all cycles of chemotherapy will be as follows:

1. **Control arm (3 drug antiemetic regimen)**

Day 1

60 mins before chemotherapy,

capsule aprepitant 125 mg PO plus

palonosetron 0.25mg IV plus

Dexamethasone 12 mg IV

Day 2 and Day 3

capsule aprepitant 80 mg PO

Written Instructions will be given to patient regarding oral intake of capsule Aprepitant for D2 and D3

2. **Experimental arm (4 drug antiemetic regimen)**

Day 1

60 mins before chemotherapy,

capsule aprepitant 125 mg PO plus

palonosetron 0.25mg IV plus

Dexamethasone 12 mg IV

Tablet Olanzapine 10 mg PO at night prior to sleep (oral and written instructions will be given to patient)

Day 2 and Day 3

capsule aprepitant 80 mg PO plus

Tablet Olanzapine 10mg PO at night prior to sleep

Oral and written Instructions will be given to patient regarding oral intake of capsule Aprepitant and tablet olanzapine for day 2 and day 3.

The length of time each subject participates in the study depends on time to first emetic episode. This may vary from after the first cycle of chemotherapy to the third cycle of chemotherapy (no emetic episodes), unless the subject no longer meets the inclusion criteria or the patient wants to exit the study.

III.Dosing schema (Figure 1)

	Study day 1	Study day 2	Study day 3
Study arm/Timing	60 minutes prior to chemotherapy	Morning and evening	Morning and evening
Control	capsule aprepitant 125 mg PO<br><br>palonosetron 0.25mg IV<br><br>Dexamethasone 12 mg IV	capsule aprepitant 80 mg (morning alone)	capsule aprepitant 80 mg (morning alone)

Experimental	capsule aprepitant 125 mg PO<br><br>palonosetron 0.25mg IV<br>Dexamethasone 12 mg IV<br><br>Tab Olanzapine 10 mg PO at night	capsule aprepitant 80 mg (morning alone)<br><br>Tab Olanzapine 10 mg PO at night	capsule aprepitant 80 mg (morning alone)<br><br>Tab Olanzapine 10 mg PO at night

5. STUDY MEDICATIONS

This study is a randomized, parallel-group, open label controlled phase 3 study making a valid comparison between a 4 drug and 3 drug antiemetic regimen for patients receiving high moderate emetogenic chemotherapeutic regimens. Subjects in the control arm are treated with the current guideline recommended antiemetic regimen. On the other hand, subjects in the active treatment arm might gain benefit compared to controls. To our knowledge, subjects will not be exposed to any additional risks other than those following the course of anti-cancer treatment and standard supportive care.

1. **Olanzapine**

Olanzapine is available as an oral medication (available as 2.5mg, 5 mg, 7.5 mg and 10mg tablets).Olanzapine blocks multiple neurotransmitter receptors including dopaminergic D1, D2, D3, D4 brain receptors, serotonergic 5-HT2a, 5-HT2c, 5-HT3, 5-HT6 receptors, catecholamine alpha1 adrenergic receptors, acetylcholine muscarinic receptors, and histamine H1 receptors. It is approved as a part of the antiemetic regimen for highly emetogenic chemotherapy (HEC), while a number of studies have shown benefit with Olanzapine in moderately emetogenic chemotherapy (MEC) as well.

I.Rationale for dosage of Olanzapine

In this study, Olanzapine will be used as once daily dosing with 10 mg tablet at night before going to sleep. The reason for this dosing is based on available data from studies showing efficacy and safety of this dosage. In the study evaluating Olanzapine in HEC by Navarri et al,

while no grade 5 toxic effects were noted, the only additional potentially significant side-effect seen was severe sedation of 5% on day 2. Besides, there was no untoward side effects.

II.Potential for adverse reactions with Olanzapine

As per the drug insert for Olanzapine, it is reported that postural hypotension, constipations, weight gain and dizziness are side effects that are commoner (at least more than double) in patients using Olanzapine as compared to placebo when used in patients with schizophrenia and bipolar disorders. However, in the study evaluating Olanzapine versus placebo in HEC, the only significantly different adverse reaction was increased sedation on D2 of protocol (5 %). Additionally, there are no known or documented drug interactions between olanzapine and dexamethasone, palonosetron or aprepitant.

2. Aprepitant

Aprepitant is available as a set of oral capsules, comprising 3 capsules of strength 125 mg (1 capsule) and 80 mg (2 capsules). It is a substance P/neurokinin 1 (NK1) receptor antagonist and is approved as a part of the antiemetic regimen for HEC, and moderately emetogenic chemotherapy (MEC) as well.

I.Rationale for dosage

The current recommendations for adult Aprepitant dosing as HEC antiemetic prophylaxis is 125mg on D1 (prior to chemotherapy) and 80mg on D2 and D3 respectively, based on published data.

II.Potential for adverse reactions with Aprepitant

Aprepitant is a standard of care for HEC and certain MEC regimens. It has been widely used since introduction and side effects which are commoner (but not statistically or clinically significantly different) when compared to ondansetron and dexamethasone combination alone are fatigue (13% vs. 12%), diarrhoea (9% vs. 8%), asthenia (7% vs 6%) and dyspepsia (6% vs. 5%) amongst others

3. Dexamethasone

Dexamethasone is available as 4 mg tablets and is part of standard of care for use as AEP

I.Rationale for dosage of Dexamethasone

Dexamethasone will be given in the dose 12 mg Day 1, with no oral dexamethasone as part of delayed antiemetic protocol. This is based on recommendations in situations when Aprepitant is being used, then delayed dexamethasone can be eliminated.

II.Potential adverse reactions, dexamethasone

The incidence of predictable adverse reactions of corticosteroids correlates with the relative potency of the drug, dosage, timing of administration, and the duration of treatment. The doses used in prevention of chemotherapy induced nausea and vomiting are high, though major adverse events will be mitigated due to the avoidance of delayed oral dexamethasone and single IV administration with chemotherapy. The potential adverse reactions are

Metabolic: Cushingoid facies. Impaired carbohydrate tolerance with increased requirement for anti-diabetic therapy. Increased appetite.

Anti-inflammatory and immunosuppressive effects: Increased susceptibility and severity of infections with suppression of clinical symptoms and signs.

Musculoskeletal: Osteoporosis, bone fractures, tendon rupture, and avascular osteonecrosis.

Fluid and electrolyte disturbances: Sodium and water retention, hypertension, potassium loss, hypokalemic alkalosis.

Neuropsychiatric: Affective disorders (e.g. irritable, euphoric, and depressed), psychotic reactions (e.g. mania, delusions, hallucinations, and aggravation of schizophrenia), behavioral disturbances, irritability, anxiety, sleep disturbances, and cognitive dysfunction including confusion and amnesia have been reported. Reactions are common (≥1/100 and <1/10).

Ophthalmic: Increased intraocular pressure, glaucoma, papilledema, posterior sub capsular cataracts, corneal or scleral thinning, exacerbation of ophthalmic viral or fungal diseases.

Gastrointestinal: Dyspepsia, peptic ulceration with perforation and hemorrhage, acute pancreatitis, esophageal ulceration and candidiasis. Abdominal distension and vomiting.

Dermatological: Impaired healing, skin atrophy, bruising, telangiectasia, striae, acne.

General: Hypersensitivity including anaphylaxis, has been reported. Leukocytosis. Thromboembolism. Myocardial rupture following recent myocardial infarction. Nausea. Malaise. Hiccups

4. Palonosetron

IV palonosetron is a part of standard AEP.

I.Rationale for dosing of palonosetron

The dose of palonosetron used in the study, 0.25, is as per the standard recommendation for its use in adults.

II.Potential adverse reactions, palonosetron

Adverse events commonly seen (>=1/100 and < 1/10) are Headache, dizziness, constipation, and diarrhoea.

Uncommon (≥1/1000 and <1/100): Hyperkaliemia, metabolic disorders, hypocalcaemia, hypokalaemia, anorexia, hyperglycemia, and decreased appetite. Anxiety and euphoric mood. Somnolence, insomnia, paranesthesia, hypersomnia, and peripheral sensory neuropathy. Eye irritation and amblyopia. Motion sickness and tinnitus. Tachycardia, bradycardia, extra systoles, myocardial ischemia, sinus tachycardia, sinus arrhythmia, and supraventricular extra systoles. Hypotension, hypertension, vein discoloration, and vein distended. Hiccups. Dyspepsia, abdominal pain, abdominal pain upper, dry mouth, and flatulence. Hyperbilirubinemia. Dermatitis allergic and pruritic rash. Arthralgia. Urinary retention and glycosuria.asthenia, pyrexia, fatigue, feeling hot, and influenza like illness. Elevated transaminases and electrocardiogram QT prolonged.

6. **Randomization**

This will be an open label parallel group randomized study.

Block randomization on a 1:1 ratio and allocation concealment will be used to eliminate bias.

The randomization code will be generated and stored by an independent person at the Clinical Research Unit, TMH.

7. I. Preparation of medicines (antiemetics) for administration

Aprepitant capsules and olanzapine tablets do not require any specific preparation prior to administration. Appropriate number of tablets of olanzapine (3 tablets) will be administered in a small bottle with the following label -

1. Protocol title
2. Allocation number
3. Content

4. Instructions for use - 1 tablet will be administered by site staff 30 min. before chemotherapy, followed by one tablet in the evening (8 p.m.). Day 2: Take one tablet in the morning (8 a.m.) AND one tablet in the evening (8 p.m.). Day 3: Take one tablet in the morning (8 a.m.) AND one tablet in the afternoon (8 p.m.).

Aprepitant is available as a kit with instructions for use explained and this will be reiterated by site staff.
IV palonosetron and IV dexamethasone will undergo preparation according to the commercial product label prior to administration.

II. Handling and storage

All study medication must be stored in a secure area with access limited to the authorized site staff and under physical conditions that are consistent with study medication -specific requirements. Clinical supplies are to be dispensed only in accordance with the protocol.

8. Documentation

The PI and study coordinator will record details of chemotherapy protocol, antiemetic medications disbursed and compliance of patients to patients.

9. Assessment of compliance

IV dexamethasone, and IV palonosetron will be administered as routine under supervision by study staff on D1. Patient Diary (see Appendix) documentation, will be explained to each patient prior to discharge from clinical site for optimal compliance and data collection. A telephone call, by the study nurse or designated site staff, on study Day 2 to Day 5, in the morning, will be conducted to ensure compliance and evaluate nausea and vomiting. An extra telephone call can be conducted if considered necessary for optimal compliance and evaluation of vomiting of that subject. A history of parallel or alternative medication history will be taken regularly.

10. Study Closure

I. Study closure

Upon completion or premature discontinuation of the study, the monitor will conduct site closure activities with the Investigator or site staff, as appropriate, in accordance with applicable regulations and GCP procedures.

II.Subject withdrawal

A subject may withdraw from the study at any time at their own request, or they may be withdrawn at the discretion of the Investigator for safety reasons or if the subject no longer meets the inclusion criteria.

If a subject is withdrawn from the study for any reason, the Investigator must make every effort to perform the following evaluations: AEs, efficacy assessments, concurrent medication, and use of rescue medication. These data will be recorded, as they comprise an essential evaluation that needs to be done prior to discharging any subject from the study. Additionally, the reason for withdrawal will be recorded in the CRF. In the event that a subject is prematurely discontinued from the study at any time due to an AE or a SAE, the procedures stated in section "Adverse events (AEs) and serious adverse events (SAEs)" and "Follow-up visit" must be followed.

III. Source documents

The subject's hospital record is the source document for the subject's baseline assessments and subsequent assessments performed during the study period. A physical copy of these documents and /or electronic records will comprise actual study records. The Patient Diary and telephonic calls will comprise the source document for efficacy measurements. The FLIE questionnaire is the source document for quality of life measurements. The adverse event form is the source document for tolerability measurements. A 'study master file' will have a copy of all the above mentioned documents.

11. Study population

I. Screening visit, patient information, informed consent

Patients receiving the below mentioned chemotherapeutic s will be screened for eligibility and asked for study participation in the outpatient clinic -

1. Modified FOLFIRI (every 2 weeks)
2. CAPIRI (every 3 weeks)
3. CAPOX (every 3 weeks)
4. Modified FOLFOX

5. Pemetrexed plus Carboplatin (every 3 weeks)
6. Paclitaxel plus Carboplatin (every 3 weeks)
7. GEMOX (Gemcitabine + Oxaliplatin) (every 2 weeks)
8. Gemcitabine plus carboplatin (weekly d1,d8,d15)

Patients will be screened from the outpatient clinics of the Medical Oncology units (Solid tumor I, GI and Solid tumor 2), treating the following tumors

1. Esophageal, gastro-esophageal and gastric cancers
2. Non-small cell lung cancers
3. Pancreatic cancers
4. Gallbladder cancers
5. Small- bowel adenocarcinomas
6. Penile cancers
7. Urinary bladder transitional cell carcinomas
8. Colorectal cancer

The information, oral and written, will be given by the Investigator or a designee. All screened patients will be recorded on the “Screening list”. Patients who meet all the inclusion criteria and none of the exclusion criteria will be recorded on the “Inclusion list” and on the “Identification list” when assigned a Patient ID-number for the study. Patients are informed about adequate time for reflection concerning study participation. Patients can return the signed informed consent form at any time after the study information is given until the day before the first course of planned chemotherapy as long as the patient feels that she has had adequate time for reflection. Patients are informed about adequate time for reflection concerning study participation, and return of the signed informed consent form will be obtained after 24 hours and within 48 hours. The subject can return the ICF before the 24 hours as long as the subject feels that she have had adequate time for reflection.

II. Patient inclusion criteria

1. Patients has a confirmed diagnosis of one of the cancers mentioned previously and is receiving one of the mentioned chemotherapy protocols mentioned previously.

2. The patient understands the nature and purpose of this study and the study procedures and has signed informed consent.
3. The patient is aged > 18 years.
4. Patients should be chemotherapy naïve.
5. The patient has a WHO Performance Status of ≤ 1.
6. Hematologic and metabolic status must be adequate for receiving planned chemotherapy, and meet the following criteria:

   Total neutrophils ≥ 1500/mm3

   Platelets ≥ 100,000/mm3

   Bilirubin ≤ 1.5 x ULN (Upper Limits of Normal)

   Aspartate aminotransferase (AST) and/or alanine aminotransferase (ALT) ≤ 3 x ULN

   GFR ≥ 50 ml/min
7. The patient is able to read, understand, and complete questionnaires and daily components of the Patient Diary for each study cycle.
8. For patients of childbearing potential, urine human chorionic gonadotropin (hCG) (urine dipstick pregnancy test) or blood hCG results must be negative at screening.
9. Has a normal baseline ECG with no QTc prolongation

III. Patient exclusion criteria

1. The patient is unable to read, understand, and complete the forms required for the study.
2. The patient is pregnant or lactating.
3. The patient has experienced emesis (i.e., vomiting and/or retching) or clinically significant nausea (defined as nausea graded as moderate or severe) in the 24 hours preceding the first dose of study medication.
4. The patient has a history active peptic ulcer disease, significant or symptomatic, acute or subacute gastrointestinal obstruction, increased intracranial pressure,severe cognitive impairment, known central nervous system disease (e.g. brain metastases/ a seizure disorder).
5. hypercalcemia, or any uncontrolled medical condition (other than malignancy) which in the opinion of the Investigator may confound the results of the study, represent another potential etiology for emesis and nausea (other than CINV) or pose an unwarranted risk to the patient.

6. The patient has a known hypersensitivity or contraindication to palonosetron, another 5-HT3 receptor antagonist, dexamethasone, aprepitant or olanzapine
7. The patient has received an investigational drug in the previous 6 months or is scheduled to receive any investigational drug other than fosaprepitant dimeglumine during the study period
8. The patient has taken/received any medication of moderate or high emetogenic potential within the 48 hours prior to the first dose of study medications. Opiate drugs for cancer pain will be permitted if the patient has been on a stable dose and has not experienced emesis or clinically significant nausea from the narcotics in the 24 hours preceding the first dose of study medication.
9. The patient has taken/received any medication with known or potential antiemetic activity within the 24-hour period prior to receiving study drugs. This is inclusive of, but not limited to 5 HT3 antagonists, metoclopramide, benzodiazepines, phenothiazines, haloperidol, oral or intravenous steroids, antihistamines, domperidone, olanzapine, antipsychotics.
10. Patient on antipsychotics or being planned to receive any of the antiphychotics, amifostine in last 3 months.
11. Patient has received concurrent abdominal radiotherapy in last 3 months or being planned to receive the same.
12. Patient is receieving or will likey to receive concurrent use of quinolone antibiotic therapy.
13. Patient with the history of chronic alcoholism.
14. Patient with cardiac arrhythmia, uncontrolled/ controlled heart failure, or acute coronary event or uncontrolled diabetes mellitus within the previous 6 months.
15. Has taken drugs which may influence medications used in the study, e.g. CYP inducers or inhibitors. This will have to be evaluated for and decision taken by PI

IV. Screening and baseline failures

If a subject experiences vomiting/retching or uncontrolled nausea at any time during the 24 hours prior to receiving study drugs, they are not eligible to receive study drugs and must be withdrawn from the study. Additionally, for subjects screened prior to the day the initial dose of study drugs is administered, eligibility will be reviewed prior to the administration of any study drugs. If there are any other changes in eligibility according to the inclusion/exclusion criteria in section 8.2. / 8.3., the subject must be withdrawn from the study. Such subjects who are

withdrawn prior to receiving study drugs should not undergo any further study-related procedures.

11. Study assessments and procedures

Overview

The study visits and procedures, e.g. blood samples, are parts of the scheduled treatments for the subject's cancer disease.

I. Demographic and baseline assessments

A signed, written informed consent form (ICF) will be obtained prior to any study specific procedures or assessments being initiated. All screening and baseline procedures will be conducted prior to the administration of the first dose of study medication and chemotherapy on Study Day 1.

Baseline details that will be collected are mentioned in clinical record form

II.Assessments in cycle 1

A cycle is a two or three-week period beginning from the time of initiation of planned chemotherapy (STUDY DAY 1) and ending on the morning of study D1 in the following cycle.

Cycle 1 Day 1 visit

Prior to initiation of D1 chemotherapy (on the day of chemotherapy administration), patient will be randomized to one of 2 treatment groups. Assessment and procedures to be performed prior to the first dose of study medication on study Day 1 will be as follows -

1. ECOG PS
2. Review of inclusion and exclusion criteria
3. Baseline assessments within the Patient Diary will be completed
4. Baseline assessments within the adverse events form will be completed.
5. Registration of how the subject is provided with rescue antiemetics and type of rescue antiemetic drug
6. Patient will complete baseline FLIE-questionnaire, and return the questionnaire to the study nurse immediately

60 minutes prior to initiation of chemotherapy on Study Day 1, subjects will be administered

1. **Control arm (3 drug antiemetic regimen)**

Day 1

60 mins before chemotherapy,

capsule aprepitant 125 mg PO plus

palonosetron 0.25mg IV plus

Dexamethasone 12 mg IV

2. **Experimental arm (4 drug antiemetic regimen)**

Day 1

60 mins before chemotherapy,

capsule aprepitant 125 mg PO plus

palonosetron 0.25mg IV plus

Dexamethasone 12 mg IV

Will all be administered by trial coordinator or nursing coordinator

Tablet Olanzapine 10 mg PO at night prior to sleep (oral and written instructions will be given to patient)

At the completion of Study Day 1 visit, subjects will be dispensed the following:

1. the appropriate study medication for Study Days 2 to 3,
2. the Patient Diary,
3. complete instructions (oral and written) for the use of each dispensed study item (oral and written)
4. Instructions on reporting of nausea as per visual analogue scale (VAS) by telephonic conversation

Study Day 2-3

Patients will self-administer drugs as explained on D1 as per the following schedule -

Control arm(3 drug antiemetic regimen)

Day 2 and Day 3

capsule aprepitant 80 mg PO

As per written Instructions given to patient on D1 regarding oral intake of capsule Aprepitant for D2 and D3

2. **Experimental arm (4 drug antiemetic regimen)**

Day 2 and Day 3

capsule aprepitant 80 mg PO plus

Tablet Olanzapine 10mg at night prior to sleep

As per written instructions given to patient on D1 regarding oral intake of capsule Aprepitant and tablet olanzapine for day 2 and day 3.

Patients in both arms will be contacted telephonically (D2 - D5) to evaluate the following information -

1. Intake of prescribed medications as per schedule
2. Reporting of vomiting in terms of number of episodes, volume
3. Assess the need for hospital visit if required
4. Reporting of nausea as per VAS
5. Need for rescue medications
6. Confirmation of planned visit on D 8- D10 as part of mid cycle evaluation

Mid-cycle evaluation (Day 7 - 10)

Patients in both arms of study will be evaluated once mandatorily (and further if required clinically) between Day 7 - Day 10 post chemotherapy as part of standard of care for patients receiving chemotherapy for the 1st time. Assessments done at this time as part of protocol will be -

1. Reconfirming intake of antiemetic medications as prescribed for D2-D3/D2-D4
2. Documenting episodes of nausea and vomiting from D1 - D7 of chemotherapy
3. Documenting nausea score as per VAS
4. Documenting need of and duration of rescue medications
5. Addressing the need for additional antiemetics if required for next cycle of chemotherapy
6. Evaluation of patient diary
7. Ensuring compliance with chemotherapy and scheduling of next visit
8. Completion of FLIE
9. questionnaire
10. Rechecking the alternative medications/parallel self-medications apart from prescribed medications.

Cycle 1 end of cycle visit and assessments in cycles 2-3

Patients will be assessed at end of cycle visit (Day 1, cycle 2). The following assessments and procedures will be performed:

• Review of the Patient Diary, Cycle 1 (Days 1-7 completed).

• Adverse event assessment.

• WHO performance status.

Post cycle 1 of chemotherapy, attempts will be made to capture information regarding nausea and vomiting in remaining cycles of chemotherapy (upto maximum of cycle 4).

III. Follow up visit and subject completion

The patient will have been assessed to have completed follow up if -

1. Attends 1st visit between D7 - D10

For the purposes of evaluating primary study objectives, the patient will have completed study upon returning for the follow up visits post cycle 1 as previously detailed. This should include return of a completed patient diary, as well as filed out the FLIE questionnaire provided during the course of the study.

IV.Antiemetic rescue medications

Definition - Rescue therapy is defined as any medication used to relieve symptoms of established nausea or vomiting and not those used for prevention of nausea or vomiting.

The Investigator will instruct subjects that they are allowed to take rescue therapy for nausea or vomiting if needed. At the time of discharge from the clinical facility on Study Day 1, subjects will document the use of rescue medication in the Patient Diary. For any antiemetic rescue medications, the name of the antiemetic, the corresponding doses, and the date of administration will be recorded in the Patient Diary.

Permitted rescue medications

The following medications will be allowed as rescue medications

1. Dexamethasone
2. Metoclopramide
3. Lorazepam
4. Olanzapine (predominantly in the 3 drug arm)
5. Phenothiazines like chlorpromazine etc.
6. Domperidone

V.Antiemetic treatment following study treatment failure

Treatment failure is defined as the occurrence of emesis post administration of antiemetics as per study protocol. Antiemetic treatment of these subjects will be as per investigator discretion.

12. Safety

Pregnancy

For females of childbearing potential, urine or blood human chorionic gonadotropin (hCG) results must be negative at screening. A pregnancy test need only be repeated if the investigator feels there is any possibility that pregnancy has occurred while the subject was being treated on protocol therapy. Pregnancies are highly improbable during the course of cancer directed

chemotherapy. In the most improbable event that a pregnancy should occur, the subject will be immediately withdrawn from the study and will receive counselling regarding nature of study medications and potential risk to fetal development.

13. Assessment of efficacy and tolerability

I. Efficacy measurements

Patient diary

Patients enrolled in the study will complete a 7 days ‘Patient diary’ that will be used to record the occurrence of emesis/retching, nausea, and the use of rescue medications daily during the assessment period. Subjects will record number of emetic episodes (vomits and nausea episodes) and using a Visual analogue scale (VAS), subjects will be instructed to complete their rating of nausea over the preceding 24 hours each morning for the 7 days following the administration of chemotherapy. The Patient Diary will be given to the subject prior to the administration of the study medication on Study Day 1 of each cycle. The study coordinator will provide instructions to the subject for completing the required sections and will familiarize the subject with the Patient diary. Patient will be required to submit the diary during planned mid cycle assessment.

Efficacy will be assessed through data collection of the following -

1. Whether or not the subject experienced any occurrence of emesis (vomiting and/or retching), and if so, the number of episodes, and time (days) to first episode.
2. Whether or not the subject experienced nausea, and if so, the subject -reported degree of nausea.(based on VAS)
3. The use of any antiemetic medications administered as rescue medications.

Assessment of toxicity will be performed by recording adverse event data using the NCI - CTCAE version 4.03.

The VAS for nausea, degree of CINV and QOL will be independently collected by non-study personnel and be considered as final for data reporting and data evaluation purposes.

Telephonic information

The patients will be contact by telephone by research coordinator on D2 - D5 post chemotherapy for

1. Confirmation of intake of prescribed antiemetic medications.
2. Assessment of degree of nausea (as per VAS) as well as details of vomiting if occurred.
3. Recounselling regarding the use of rescue medications.

4. Confirmation of details of next clinical visit

II.Nausea scales

Nausea will be measured on a scale of 1-100 using a visual analogue scale. Events will be described as follows -

1. No nausea is defined as a VAS score of less than 5 mm on the 100-mm scale.
2. A patient will be considered to have had acute nausea or acute emesis if nausea (VAS > = 5 mm), or at least one episode of vomiting was reported during the first 24 h after start of chemotherapy. The occurrence within 24 hours of nausea or vomiting will be considered as an episode of acute nausea or emesis
3. Any episode of nausea and/or vomiting thereafter (beyond 24 hours post chemotherapy up to 5 days after chemotherapy will be considered as delayed nausea or vomiting.

III.Tolerability measurements

Adverse events and serious adverse events

The Investigator will detect and document events meeting the criteria and definition of an AE or SAE, as provided in this protocol. Investigators will use the NCI Common Terminology Criteria for Adverse Events (CTCAE), Version 4.03 for assessing the severity of adverse events; these data will be collected in the adverse events record.

Definition of an adverse event (AE)

An ADVERSE EVENT (AE) is any untoward medical occurrence in a patient or clinical investigational subject administered a pharmaceutical product and which does not necessarily have a causal relationship with this treatment. An AE can therefore be any unfavourable or unintended sign (including an abnormal laboratory finding), symptom, or disease temporally associated with the use of a medicinal investigational product, whether or not considered related to the medicinal product (see below).

Adverse events include the following:

1. All suspected adverse drug or device reactions
2. All reactions from drug or device – overdose, abuse, withdrawal, sensitivity, toxicity or failure of expected pharmacological action (if appropriate)
3. Apparently unrelated illnesses, including the worsening (severity, frequency) of pre-existing illnesses

4. Injury or accidents.
5. Abnormalities in physiological testing or physical examination that require clinical intervention or further investigation (beyond ordering a repeat examination)
6. Laboratory abnormalities that require clinical intervention or further investigation (beyond ordering a laboratory test).
7. Any untoward event that occurs after the protocol-specified reporting period which the Investigator believes may be related to the drug or device.

AEs are not required to be reported unless they meet SAE criteria.

Definition of a serious adverse event (SAE)

A SERIOUS ADVERSE EVENT (SAE) is any untoward medical occurrence that at any dose that results in

1. Death,
2. is life-threatening (i.e. the subject is at risk of death at the time of the event),
3. requires inpatient hospitalization or prolongation of existing hospitalization,
4. Results in persistent or significant disability or incapacity,
5. Is a congenital anomaly/birth defect,
6. Other important medical events which, in the opinion of the investigator, are likely to become serious if untreated, or as defined in the protocol

NOTES:

The term “life-threatening” in the definition of “serious” refers to an event in which the patient was at risk of death at the time of the event; it does not refer to an event which hypothetically might have caused death if it were more severe.

Important medical events which may not be immediately life-threatening or result in death or hospitalization but which may jeopardize the patient or may require intervention to prevent one of the listed outcomes in the definition above should also be considered serious.

Definition of AR, SAR, and SUSAR

Adverse reaction (AR):

• Is an adverse event considered related to the medicinal product.

Serious adverse reaction (SAR):

• Is a serious adverse event considered related to the medicinal product

A **SUSPECTED UNEXPECTED SERIOUS ADVERSE REACTION (SUSAR)**is an SAE that is related to the drug or device and is unexpected (i.e. not listed in the investigator brochure or approved Product Information; or is not listed at the specificity or severity that has been observed; or is not consistent with the risk information described in the Subject Information Sheet and Informed Consent Form or elsewhere in the protocol. (FDA, Safety Reporting Requirements for INDs and BA/BE Studies, draft guidance, September 2010)).

An event is causally related if there is a reasonable possibility that the drug [intervention] caused the AE, i.e. there is evidence to suggest a causal relationship between the drug and the event (FDA, Safety Reporting Requirements for INDs and BA/BE Studies, draft guidance, September 2010).

For the purposes of this study, the following adverse events are not reported as SAEs:

-hospitalization or death as a result of or related to disease progression.

14. Disease-related events and/or disease-related outcomes not qualifying as SAEs / SARs

During the 168 hours (7 days) assessment period, episodes of nausea or vomiting that result in the administration of rescue medication are to be considered treatment failures rather than AEs / ARs or SAEs / SARs. Also during this assessment period, visits to the emergency department or hospital admissions that are solely attributable to such treatment failures and result in the administration of rescue medication or related therapy (e.g., intravenous fluids) also do not qualify as AEs / ARs or SAEs / SARs, and are not to be reported as such episodes.

Throughout the study, any event which is part of the natural course of the underlying neoplasm (i.e., disease progression), and is considered by the Investigator to be directly caused by the subject's neoplasm, does not need to be reported as an SAE. In addition, any death due to progression of the underlying neoplasm also is to be excluded as an SAE.

15. Clinical laboratory abnormalities and other abnormal assessments as AEs / ARs and SAEs / SARs

Abnormal laboratory findings (e.g., clinical chemistry, hematology) or other abnormal assessments that are judged by the Investigator as clinically significant will be recorded as AEs / ARs or SAEs / SARs if they meet the definition of an AE / AR or SAE / SAR. Clinically significant abnormal laboratory findings or other abnormal assessments that are detected during the study or are present at baseline and significantly worsen following the start of the study will

be reported as AEs or SAEs. However, clinically significant abnormal laboratory findings or other abnormal assessments that are associated with the cancer disease or the chemotherapy, unless judged by the Investigator as more severe than expected for the subject's condition, or that are present or detected at the start of the study and do not worsen, will not be reported as AEs or SAEs. The Investigator will exercise his or her medical and scientific judgment in deciding whether an abnormal laboratory finding or other abnormal assessment is clinically significant.

16. Recording and reporting of AE, AR, SAE, SAR, and SUSAR

AEs / ARs and SAEs / SARs that occur following the first dose of study medication until 8 days following the last dose of study medication, regardless of the relationship to study medication, will be collected and recorded on the subject's CRF.

From the time a patient consents to participate in the study until she has completed the study (including the follow-up period), all SAEs / SARs / SUSARs assessed as related to study participation will be reported to IRB/IEC

Nausea and vomiting that occur during the 168 hours (7 days) following administration of chemotherapy will be documented in the Patient Diary during each cycle of therapy and should not be recorded as adverse events.

SAE will be reported for the above events during the active period of the trial.SAE/SUSAR/SAR reports will be forwarded to the IEC in the IEC approved format within 24 hours.

17. Quality of life measurements and sample collection

FLIE questionnaire

Quality of Life (QoL) data will be analyzed to determine if there are treatment differences among the two treatment groups both before treatment (baseline) and during treatment. The Functional Living Index-Emesis (FLIE) questionnaire will be used for this study.

The FLIE questionnaire will be administered at the following time points -

1. Prior to administration of cycle 1
2. Mid - cycle assessment - C1 D7 - D10

As previously detailed, the FLIE questionairre will be administered and collected by non-study personnel/ non study interventional team for the purposes of study. The investigators will be blinded to the QOL till the time of completion of study and data analysis.

18. Concomitant treatment

I. Chemotherapy

The following drugs in the given schedules will be administered -

1. Modified FOLFIRI (5 Fluorouracil + Leucovorin + Irinotecan) (biweekly)
   5 Fluorouracil 2400 mg/m2 intravenous infusion over 46-48 hours (diluted in 0.9% normal saline (NS)
   Irinotecan 180 mg/m2 intravenous infusion over 90 minutes (diluted in 0.9% normal Saline or D5W)
   Leucovorin 400 mg intravenous over 2 hours (prior to 5 Fluorouracil infusion)
2. CAPIRI (Capecitabine + Irinotecan) (every 3 weeks)
   Tablet Capecitabine 1700 mg/m2/day in two divided doses per oral for 14 days, then 7 days off
   Irinotecan 200 mg/m2 intravenous infusion over 90 minutes (diluted in 0.9% normal Saline or D5W)
3. CAPOX (Capecitabine + Oxaliplatin) (every 3 weeks)
   Tablet Capecitabine 2000 mg/m2/day in two divided doses per oral for 14 days, then 7 days off
   Oxaliplatin 130 mg/m2 intravenous infusion over 120 - 360 minutes (diluted in D5W)
4. Modified FOLFOX (5 Fluorouracil + Leucovorin + Oxaliplatin) (biweekly)
   5 Fluorouracil 2400 mg/m2 intravenous infusion over 46-48 hours (diluted in 0.9% normal saline (NS)
   Oxaliplatin 85 mg/m2 intravenous infusion over 120 - 360 minutes (diluted in D5W)
   Leucovorin 400 mg intravenous over 2 hours (prior to 5 Fluorouracil infusion)
5. Pemetrexed plus Carboplatin (every 3 weeks)
   Pemetrexed 500 mg/m2 intravenous over 10 mins (diluted in 0.9% normal saline)
   Carboplatin (AUC - 5 or AUC - 6) intravenous over 30 minutes
6. Paclitaxel plus Carboplatin (every 3 weeks)
   Paclitaxel 175 - 225 mg/m2 intravenous over 3 hours (diluted in 0.9% normal saline)
   Carboplatin (AUC - 5 or AUC - 6) intravenous over 30 minutes
7. GEMOX (Gemcitabine + Oxaliplatin) (every 2 weeks)
   Gemcitabine - 1000mg/m2 intravenous over 30 minutes (diluted in 0.9% normal saline)
   Oxaliplatin 85 mg/m2 intravenous infusion over 120 - 360 minutes (diluted in D5W)

Imp - Patients receiving the above mentioned regimens and drugs alone will be recruited in study. Full doses of both drugs individually should be used for entry into study.

Patients receiving reduced doses of the drug cannot be included in study, though dose reductions/modifications can be carried out later for patients continuing chemotherapy.

II.Prohibited drugs

The following drugs will be prohibited for use by subjects in the study -

1. Any investigational drug in the previous 30 days, or during the study period.
2. Any medication with known or potential antiemetic activity within the 24-hour period prior to receiving study drug. This includes, but is not limited to: 5-HT3 receptor antagonists (e.g., ondansetron, granisetron, palonosetron (7 days prior)); benzamide/benzamide derivatives (e.g., metoclopramide, alizapride); benzodiazepines (except if the patient is receiving such medication for sleep and has been on a stable dose for at least 7 days prior to the first dose of study medication; however, lorazepam is prohibited); phenothiazines (e.g., prochlorperazine, promethazine, fluphenazine, perphenazine, thiethylperazine, chlorpromazine, metopimazine); butyrophenones (e.g., haloperidol, droperidol, domperidone); corticosteroids (e.g., dexamethasone, methylprednisolone, prednisone; with the exception of topical steroids for skin disorders and inhaled steroids for respiratory disorders); anticholinergics (e.g., scopolamine); antihistamines (e.g., cyclizine, hydroxyzine, diphenhydramine); cannabinoids, and mirtazapine.
3. Any prophylactic antiemetic medications (other than study medication) after initiation of chemotherapy and until completion of the study period. Antiemetic medications given during this time should only be given as rescue medications.

Medications which are inhibitors or inducers of CYP3A4 should be excluded for a minimum of 7 days prior and 3 days post the first dose of antiemetics, unless they are absolutely essential as per PI discretion.

Non-drug therapies

Any non-drug therapies (e.g., ginger preparations, curcumin) to treat emetic episodes are discouraged. However, in the event that such non-drug therapies are employed, the details of such use will be appropriately documented in the subject's CRF.

19. Data analysis

I. Hypothesis

The primary objective of this study is to compare an antiemetic regimen consisting of aprepitant, palonosetron, dexamethasone and olanzapine (olanzapine regimen) with an antiemetic regimen consisting of aprepitant, palonosetron and dexamethasone (non-olanzapine regimen) with respect to efficacy (sustained no emesis rate) in the course of first cycle of chemotherapy. A primary hypothesis will be tested:

• Null hypothesis: There is no difference in the 'complete response (CR) rates' following first cycle of chemotherapy between the olanzapine containing regimen and the control regimen;

$H0 : p0 = p1$

• Alternative hypothesis: The CR rates in the two treatment arms differ;

$H1 : p0 \neq p1$

where p0 and p1 represent the proportion of 'no emesis' rate during first cycle of chemotherapy.

II.Study design and statistical considerations

Assuming that 75% of patients in the non-olanzapine arm will have complete response (CR), we will require 560 patients (280 patients per arm) to show an improvement of CR rates of 10% (CR rates of 85%) with olanzapine containing arm. This is based on a two sided alpha of 5% and power of 80%, assuming 10% attrition rates. The accrual of patients will be in a 1:1 ratio uniformly over 3 years followed by 6 months for follow up.

III. Data analysis considerations

Study subject data will be collected by the Investigator or a designee using the CRF or/and eCRF. Study subject data necessary for analysis and reporting will be entered into a validated statistical software

IV.Treatment endpoints - primary and secondary

The primary endpoint of interest between the two arms of antiemetics is the complete response (CR) rates post 1st cycle of chemotherapy. This is the basis for statistical considerations as mentioned above.

Secondary endpoints

1. Sustained CR rates - comparison between the 2 arms in terms of CR over cycles 1-3
2. 'No emesis rates' between the 2 arms for cycle 1 individually and cycles 1-3 cumulatively
3. No significant nausea rates between the 2 arms for cycle 1 individually and cycles 1-3 cumulatively
4. QOL comparisons using FLIE questionnaire between the 2 arms

5. To compare tolerance and side effects with both regimens

20. Analytical plan

I.Assessment windows

Endpoints will be assessed during mid cycle assessment post cycle 1 of chemotherapy (D7 - D10), and prior to second and third cycles of chemotherapy.

II.Efficacy analysis

Analysis of the study will be conducted when all patients enrolled in the study have completed at least 3 cycles of chemotherapy with requisite antiemetic medications as per arm of study.

Primary and secondary efficacy analysis

Nonparametric statistics and the log rank test, will be used to calculate the CR rates as well as other secondary endpoints (comparison of time to event in the two treatment groups). A comparison of the proportions of subjects with AEs and SAEs in the two treatment groups will also be done.

III.Tolerability analysis

Adverse events

The incidence of adverse events up to and including the completion of the study (end of cycle 3) visit will be presented. The adverse events will be categorized according to the terms used in the CTCAE, v4.03. The following summaries will be presented:

- Subjects who experience any adverse events.
- Subjects who experience drug-related adverse events (adverse reactions). Drug related is defined by the Investigator as having a reasonable possibility that the AE may have been caused by the study medications.
- Subjects who experience any serious adverse events.
- Subjects who withdraw from the study due to an adverse event

IV.Statistical setting deviations

Any deviation, (which is however attempted not to occur), from the original statistical setting will be indicated in the clinical study report and will be reported as post hoc analysis.

21. Conduct of study

I. Ethical considerations

Emesis is an unwanted by-product of chemotherapy and all attempts should be made to reduce the incidence of emesis to as minimal as possible. This coupled with the effects of nausea on patients, which is often underrated, means that there is always a need to evaluate mechanisms to reduce these side-effects. The suggested drugs to be used in the study have been commonly

used in multiple studies and in many patients as antiemetics with no unexpected side-effects. The potential benefits of increased antiemetic use outweigh the possible minimal side - effects associated with the use of these drugs

Besides having a reduction in CINV, decreased nausea and vomiting also has an impact on quality of life and we hope to assess this aspect via the FLIE QOL questionnaires.

All patients will be informed of all aspects of the study and will be enrolled into study only after a fully informed consent. The study will only start after approval from the IEC/IRB is obtained and will be conducted as per the principles of the Declaration of Helsinki guidelines.

II. Records retention

Following closure of the study, the Investigator will maintain all site study records in a safe and secure location. The records will be maintained to allow easy and timely retrieval, when needed (e.g., audit or inspection), and, whenever feasible, to allow any subsequent review of data in conjunction with assessment of the facility, supporting systems, and staff.

The time period for retaining these records will be fifteen years after closure of the study.

III.Data management

The data collection tool for this study will be the subject's medical record, registration form, adverse events forms, Patient Diaries, and FLIE-questionnaires. All data will be entered and maintained as hard copy as well as in electronic format. Original data collection tools will be retained by the Investigators

IV. Missing data

In the clinical study report a statement will be given concerning missing data, unused data, and false data.

22. Financing and assurances

The financing for the study will be applied for from intramural as well as extramural sources after IRB/IEC approval of study

23. Publications

Post IRB/IEC approval, the trial protocol will be registered with CTRI. The results of the study will be evaluated for presenting in national conferences and international conferences as well as potential publication in peer-reviewed journals.

24. References

1. Bloechl-Daum B, Deuson RR, Mavros P, Hansen M, Herrstedt J. Delayed nausea and vomiting continue to reduce patients' quality of life after highly and moderately emetogenic chemotherapy despite antiemetic treatment. J Clin Oncol Off J Am Soc Clin Oncol. 2006 Sep 20; 24(27):4472–8.

2. Hesketh PJ, Kris MG, Grunberg SM, Beck T, Hainsworth JD, Harker G, et al. Proposal for classifying the acute emetogenicity of cancer chemotherapy. J Clin Oncol. 1997 Jan 1; 15(1):103–9.

3. Hesketh PJ, Bohlke K, Lyman GH, Basch E, Chesney M, Clark-Snow RA, et al. Antiemetics: American Society of Clinical Oncology Focused Guideline Update. J Clin Oncol. 2016 Feb 1; 34(4):381–6.

4. Yahata H, Kobayashi H, Sonoda K, Shimokawa M, Ohgami T, Saito T, et al. Efficacy of aprepitant for the prevention of chemotherapy-induced nausea and vomiting with a moderately emetogenic chemotherapy regimen: a multicenter, placebo-controlled, double-blind, randomized study in patients with gynecologic cancer receiving paclitaxel and carboplatin. Int J Clin Oncol. 2016 Jun; 21(3):491–7.

5. Hesketh PJ, Schnadig ID, Schwartzberg LS, Modiano MR, Jordan K, Arora S, et al. Efficacy of the neurokinin-1 receptor antagonist rolapitant in preventing nausea and vomiting in patients receiving carboplatin-based chemotherapy. Cancer. 2016 Aug 1; 122(15):2418–25.

6. Hesketh PJ, Kris MG, Basch E, Bohlke K, Barbour SY, Clark-Snow RA, et al. Antiemetics: American Society of Clinical Oncology Clinical Practice Guideline Update. J Clin Oncol. 2017 Jul 31; 35(28):3240–61.

7. Hesketh PJ, Grunberg SM, Gralla RJ, Warr DG, Roila F, de Wit R, et al. The Oral Neurokinin-1 Antagonist Aprepitant for the Prevention of Chemotherapy-Induced Nausea and Vomiting: A Multinational, Randomized, Double-Blind, Placebo-Controlled Trial in Patients Receiving High-Dose Cisplatin—The Aprepitant Protocol 052 Study Group. J Clin Oncol. 2003 Nov 15; 21(22):4112–9.

8. Schmoll HJ, Aapro MS, Poli-Bigelli S, Kim H-K, Park K, Jordan K, et al. Comparison of an aprepitant regimen with a multiple-day ondansetron regimen, both with dexamethasone, for antiemetic efficacy in high-dose cisplatin treatment. Ann Oncol. 2006 Jun 1; 17(6):1000–6.

9. Navari RM, Qin R, Ruddy KJ, Liu H, Powell SF, Bajaj M, et al. Olanzapine for the Prevention of Chemotherapy-Induced Nausea and Vomiting. N Engl J Med. 2016 Jul 14; 375(2):134–42.

10. Navari RM, Qin R, Ruddy KJ, Liu H, Powell SF, Bajaj M, et al. Olanzapine for the prevention of chemotherapy-induced nausea and vomiting (CINV) in patients receiving highly emetogenic chemotherapy (HEC): Alliance A221301, a randomized, double-blind, placebo-controlled trial. J Clin Oncol. 2015 Oct 10; 33(29_suppl):176–176.

11. Mizukami N, Yamauchi M, Koike K, Watanabe A, Ichihara K, Masumori N, et al. Olanzapine for the prevention of chemotherapy-induced nausea and vomiting in patients receiving highly or moderately emetogenic chemotherapy: a randomized, double-blind, placebo-controlled study. J Pain Symptom Manage. 2014 Mar; 47(3):542–50.

12. Chelkeba L, Gidey K, Mamo A, Yohannes B, Matso T, Melaku T. Olanzapine for chemotherapy-induced nausea and vomiting: systematic review and meta-analysis. Pharm Pract [Internet]. 2017; 15(1).

13. Chiu L, Chiu N, Chow R, Zhang L, Pasetka M, Stinson J, et al. Olanzapine for the prophylaxis and rescue of chemotherapyinduced nausea and vomiting (CINV): a retrospective study. Ann Palliat Med. 2016 Jul; 5(3):172–8.
