# Supplementary material for: Olanzapine as Antiemetic Prophylaxis in Moderately Emetogenic Chemotherapy: A Phase 3 Randomized Clinical Trial
Source: JAMA Netw Open. 2024 Aug 6;7(8):e2426076. doi: 10.1001/jamanetworkopen.2024.26076 (PMC11304110; doi:10.1001/jamanetworkopen.2024.26076)
Supplement: Supplement 2. — eTable 1. End Points by Frequencies and Proportions According to the Study Group in the Second Cycle of Chemotherapy eTable 2. End Points by Frequency and Proportions According to the Study Group in the Third Cycle of Chemotherapy eTable 3. End Points According to Study Group and CINV Risk Score [file jamanetwopen-e2426076-s002.pdf]

Supplementary Online Content

Ostwal V, Ramaswamy A, Mandavkar S, et al. Olanzapine as antiemetic prophylaxis in moderately emetogenic chemotherapy: a phase 3 randomized clinical trial. *JAMA Netw Open*. 2024;7(8):e2426076. doi:10.1001/jamanetworkopen.2024.26076

eTable 1. End Points by Frequencies and Proportions According to the Study Group in the Second Cycle of Chemotherapy

eTable 2. End Points by Frequency and Proportions According to the Study Group in the Third Cycle of Chemotherapy

eTable 3. End Points According to Study Group and CINV Risk Score

This supplementary material has been provided by the authors to give readers additional information about their work.

© 2024 Ostwal V et al. *JAMA Network Open*.

eTable 1. End points by frequencies and proportions according to the study group in second cycle of chemotherapy

Variable	Olanzapine (N=274)	Observation (N=270)	Total (N=544)	P value*
Complete response	262 (96)	243 (90)	505 (93)	0.011
Nausea control	266 (97)	255 (94)	521 (96)	0.127
Vomiting control	267 (97)	256 (95)	523 (96)	0.111
CINV control	262 (96)	245 (91)	507 (93)	0.024

*p value obtained by chi-square test

© 2024 Ostwal V et al. *JAMA Network Open*.

eTable 2. End points by frequency and proportions according to the study group in the third cycle of chemotherapy

Variable	Olanzapine (n=274)	Observation (N=270)	Total (N=544)	P value*
Complete response	270 (99)	259 (96)	529 (97)	0.063
Nausea control	269 (98)	261 (97)	530 (97)	0.267
Vomiting control	271 (99)	263 (97)	534 (98)	0.194
CINV control	269 (98)	257 (95)	524 (96)	0.051

*p value obtained by chi-square test

© 2024 Ostwal V et al. *JAMA Network Open*.

eTable 3. End points according to study group and CINV risk score

Variable	Olanzapine group (experimental) (n=262)		Observation group (standard) (n=253)		P value
	N	%	N	%	
Low risk					
• Complete response	184/202	91	151/180	84	0.042
• CINV control	190/202	94	152/180	84	0.002
High risk					
• Complete response	53/60	88	60/73	82	0.465
• CINV control	55/60	92	62/73	85	0.290

© 2024 Ostwal V et al. *JAMA Network Open*.
