# Supplementary material for: Olanzapine as Antiemetic Prophylaxis in Moderately Emetogenic Chemotherapy: A Phase 3 Randomized Clinical Trial
Source: JAMA Netw Open. 2024 Aug 6;7(8):e2426076. doi: 10.1001/jamanetworkopen.2024.26076 (PMC11304110; doi:10.1001/jamanetworkopen.2024.26076)
Supplement: Supplement 3. — Data Sharing Statement [file jamanetwopen-e2426076-s003.pdf]

Data Sharing Statement

Ostwal. Olanzapine as Antiemetic Prophylaxis in Moderately Emetogenic Chemotherapy. *JAMA Netw Open*. Published August 06, 2024. doi:10.1001/jamanetworkopen.2024.26076

Data
Data available: Yes
Data types: Deidentified participant data
How to access data: Deidentified data after appropriate request and approval of the IEC and regulatory bodies
When available: With publication

Supporting Documents
Document types: None

Additional Information
Who can access the data: researchers whose proposed use of the data has been approved
Types of analyses: meta analysis
Mechanisms of data availability: after approval of a proposal
